# Anti-Atopic Dermatitis Activity of *Epi*-Oxyzoanthamine Isolated from Zoanthid

**DOI:** 10.3390/md21080447

**Published:** 2023-08-12

**Authors:** Chieh-Chen Huang, Yuan-Hsin Lo, Yu-Jou Hsu, Yuan-Bin Cheng, Chia-Chi Kung, Cher-Wei Liang, Der-Chen Chang, Kang-Ling Wang, Chi-Feng Hung

**Affiliations:** 1 School of Medicine, Fu Jen Catholic University, New Taipei City 242, Taiwan; m001017@ms.skh.org.tw (C.-C.H.); a00781@fjuh.fju.edu.tw (Y.-H.L.); drpainkung@gmail.com (C.-C.K.); 085027@mail.fju.edu.tw (C.-W.L.); 2 Department of Dermatology, Shin Kong Wu Ho-Su Memorial Hospital, Taipei 111, Taiwan; 3 Department of Dermatology, Fu Jen Catholic University Hospital, Fu Jen Catholic University, New Taipei City 242, Taiwan; 4 PhD Program in Pharmaceutical Biotechnology, Fu Jen Catholic University, New Taipei City 24205, Taiwan; 411138028@mail.fju.edu.tw; 5 Department of Marine Biotechnology and Resources, National Sun Yat-Sen University, Kaohsiung 804, Taiwan; jmb@mail.nsysu.edu.tw; 6 Department of Anesthesiology, Fu Jen Catholic University Hospital, Fu Jen Catholic University, New Taipei City 242, Taiwan; 7 Department of Pathology, Fu Jen Catholic University Hospital, Fu Jen Catholic University, New Taipei City 242, Taiwan; 8 Department of Mathematics and Statistics, Department of Computer Science, Georgetown University, Washington, DC 20057, USA; chang@georgetown.edu; 9 Division of Metabolism and Endocrinology, Department of Internal Medicine, Taoyuan Armed Forces General Hospital, Taoyuan 325, Taiwan; 10 School of Pharmacy, Kaohsiung Medical University, Kaohsiung 80708, Taiwan

**Keywords:** atopic dermatitis, inflammation, keratinocyte, *epi*-oxyzoanthamine, zoanthid

## Abstract

Atopic dermatitis (AD, eczema) is a condition that causes dry, itchy, and inflamed skin and occurs most frequently in children but also affects adults. However, common clinical treatments provide limited relief and have some side effects. Therefore, there is a need to develop new effective therapies to treat AD. *Epi*-oxyzoanthamine is a small molecule alkaloid isolated from Formosan zoanthid. Relevant studies have shown that zoanthamine alkaloids have many pharmacological and biological activities, including anti-lymphangiogenic functions. However, there are no studies on the use of *epi*-oxyzoanthamine on the skin. In this paper, *epi*-oxyzoanthamine has been shown to have potential in the treatment of atopic dermatitis. Through in vitro studies, it was found that *epi*-oxyzoanthamine inhibited the expression of cytokines in TNF-α/IFN-γ-stimulated human keratinocyte (HaCaT) cells, and it reduced the phosphorylation of MAPK and the NF-κB signaling pathway. Atopic dermatitis-like skin inflammation was induced in a mouse model using 2,4-dinitrochlorobenzene (DNCB) in vivo. The results showed that *epi*-oxyzoanthamine significantly decreased skin barrier damage, scratching responses, and epidermal hyperplasia induced by DNCB. It significantly reduced transepidermal water loss (TEWL), erythema, ear thickness, and spleen weight, while also increasing surface skin hydration. These results indicate that *epi*-oxyzoanthamine from zoanthid has good potential as an alternative medicine for treating atopic dermatitis or other skin-related inflammatory diseases.

## 1. Introduction

Atopic dermatitis, also known as eczema, is a chronic inflammatory relapsing skin disease. It is one of the most common skin diseases affecting infants and young children [1]. About half of children with atopic dermatitis will develop allergic rhinitis, asthma, allergic conjunctivitis, etc. [2]. Allergies or abnormal immune function are currently believed to be important factors causing atopic dermatitis, but the true, underlying cause is not yet clear [3]. Patients have skin allergic reactions to many allergens in the environment, such as dust mites and food, and elevated levels of immunoglobulin E (IgE) [4] and eosinophilic leukocytes [5] are found in the blood of many patients [3]. Notably, itching and dry skin are the main clinical symptoms [6]. Topical steroids are currently the main treatment for atopic dermatitis. However, long-term use of topical steroids can cause skin thinning and discoloration [7]. Oral antihistamines can also be used for severe itching. Yet, it should be noted that the first-generation antihistamines are prone to the side effect of drowsiness [6], limiting the overall benefit of their use. The onset of atopic dermatitis involves multiple mechanisms, and a small number of particularly severe atopic dermatitis may even require doctors to prescribe biological agents [8] or immunosuppressants for treatment. The discovery of these biological agents and oral targeted small-molecule drugs are typically very helpful to patients as they have good therapeutic effects and minimal side effects [9]. Unfortunately, the high cost of these treatments is a strong barrier and limits patient use. Therefore, it is a medical necessity to develop an atopic dermatitis treatment with few side effects at a low price.

Human beings have been using Chinese herbal medicine for thousands of years [10]. Most scientists have discovered new drugs or compound structures from land. However, with the extensive research and development by scientists, the discovery of new structural natural components in terrestrial plants has become more and more difficult. Many scientists hope to find them in the ocean [11]. The ocean area accounts for more than half of the earth’s surface area. It is not only the origin of life on the earth, but also the most abundant treasure house of biological resources, including hundreds of thousands to millions of lower marine organisms and microorganisms. The ocean may also produce many chemical substances with special structures and significant biological activities depending on the geographical location, ocean currents, and depths [11,12]. In the past, many compounds have been found to have anti-cancer [13], anti-hyperlipidemia [14], anti-viral [15], pain-relieving [16], and anti-dementia effects [17]. Several of these candidates have been marketed or are in clinical trials [3].

Zoanthid is a kind of sessile marine invertebrate that is regarded as a good source of marine natural products. Zoanthenamine alkaloids are the predominant secondary metabolites of zoanthid. More than 70 of this type of compound have been identified, and the majority contain an azepane moiety [18]. These non-aromatic alkaloids demonstrate various pharmacological effects, including anti-inflammatory [19], anti-cancer [20], neuroprotective [21], antiosteoporosis [22], and antimetastatic activities [23]. However, the effect of this type of compound on atopic dermatitis is still unknown. Therefore, this article will leverage in vivo and in vitro experiments to show that *epi*-oxyzoanthamine among the zoanthamine compounds is a very good choice for atopic dermatitis.

## 2. Results

### 2.1. Effect of Epi-Oxyzoanthamine on HaCaT Cell Viability

**Figure 1:**
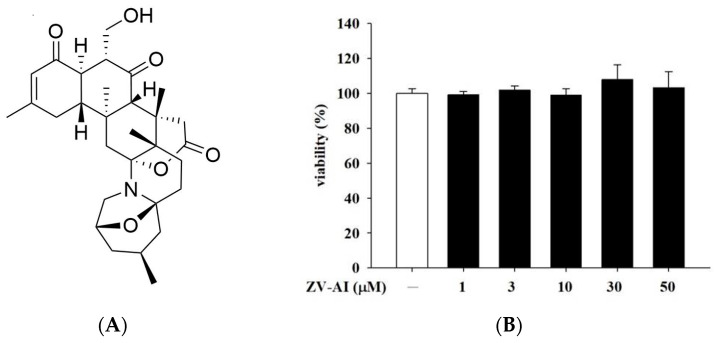
(**A**) The chemical structure of *epi*-oxyzoanthamine. (**B**) Effects of *epi*-oxyzoanthamine (ZV-AI) on cell viabilities of keratinocytes. HaCaT cells were pretreated with different concentrations of *epi*-oxyzoanthamine for 24 h.

At the beginning of the experiment, in order to prove the safety of the *epi*-oxyzoanthamine (Figure 1A), whether the toxicity of the *epi*-oxyzoanthamine is proved by cell viability test is suitable for subsequent related in vitro or in vivo studies. Thiazolyl blue tetrazolium bromide (MTT) assay was used to evaluate the cell viability. After the cells were administered for 1 h from a low dose of 1 μM to a high dose of 50 μM, we still did not find any toxicity of *epi*-oxyzoanthamine to the cells (Figure 1B). Therefore, experiments were conducted to investigate the mechanism of action of *epi*-oxyzoanthamine against atopic dermatitis in this concentration range.

### 2.2. The Anti-Inflammatory Effect of Epi-Oxyzoanthamine in Tumor Necrosis Factor-α (TNF-α)/Interferon-γ (IFN-γ)-Induced Inflammation in HaCaT Cells

**Figure 2:**
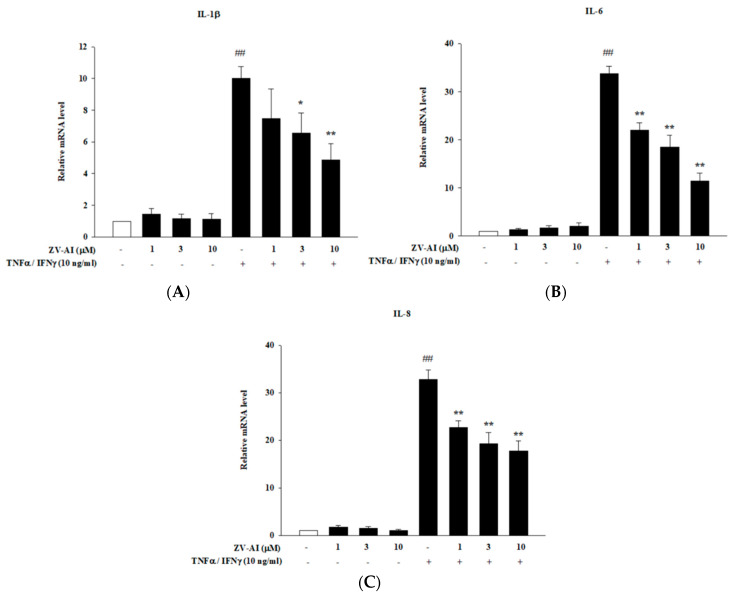
Effects of *epi*-oxyzoanthamine (ZV-AI) on the expression of correlated cytokines mRNA ((**A**): IL-1β, (**B**): IL-6, and (**C**): IL-8) after stimulation of TNF-α/IFN-γ. HaCaT cells were pretreated with different concentrations of *epi*-oxyzoanthamine for 1 h, and then the cells were treated with TNF-α/IFN-γ for 1 h. ## *p* < 0.01 compared with the no-treatment group; * *p* < 0.05 and ** *p* < 0.01 compared with the TNF-α/IFN-γ-induced group.

The increase of pro-inflammatory cytokines is closely related to the etiology of atopic dermatitis. Many studies have shown that IFN-γ and TNF-α stimulate keratinocytes to induce signaling pathways involved in pro-inflammatory responses. Therefore, this model is often used as an in vitro test method for skin anti-inflammatory effectiveness [24,25]. It is shown in Figure 2 that once these pro-inflammatory cytokines (TNF-α and IFN-γ) are added, the formation of intracellular cytokine mRNA is significantly increased. These cytokines include IL-1β, IL-6, and IL-8. Moreover, there was a direct relationship between the concentrations of *epi*-oxyzoanthamine (1–10 μM) and the inhibitory ability of the cytokines: as the drug concentration increased, so did the inhibitory ability (Figure 2A–C).

### 2.3. Effects of Epi-Oxyzoanthamine on Phosphrylation of MAPK Pathway in HaCaT Cells

**Figure 3:**
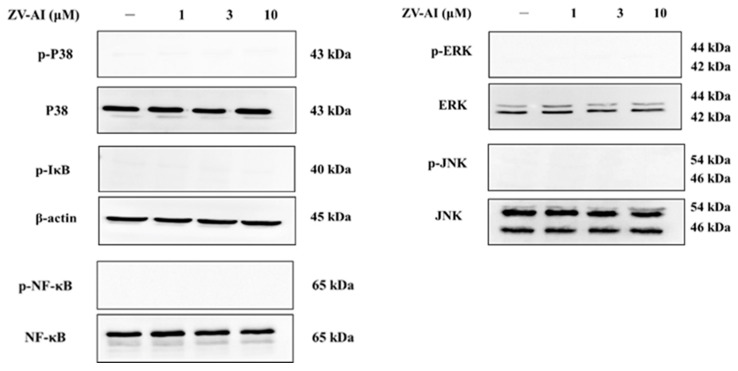
Effect of *epi*-oxyzoanthamine (ZV-AI) on phosphorylation of MAP kinase and transcription factor. HaCaT cells were pretreated with different concentrations of *epi*-oxyzoanthamine for 1 h.

**Figure 4:**
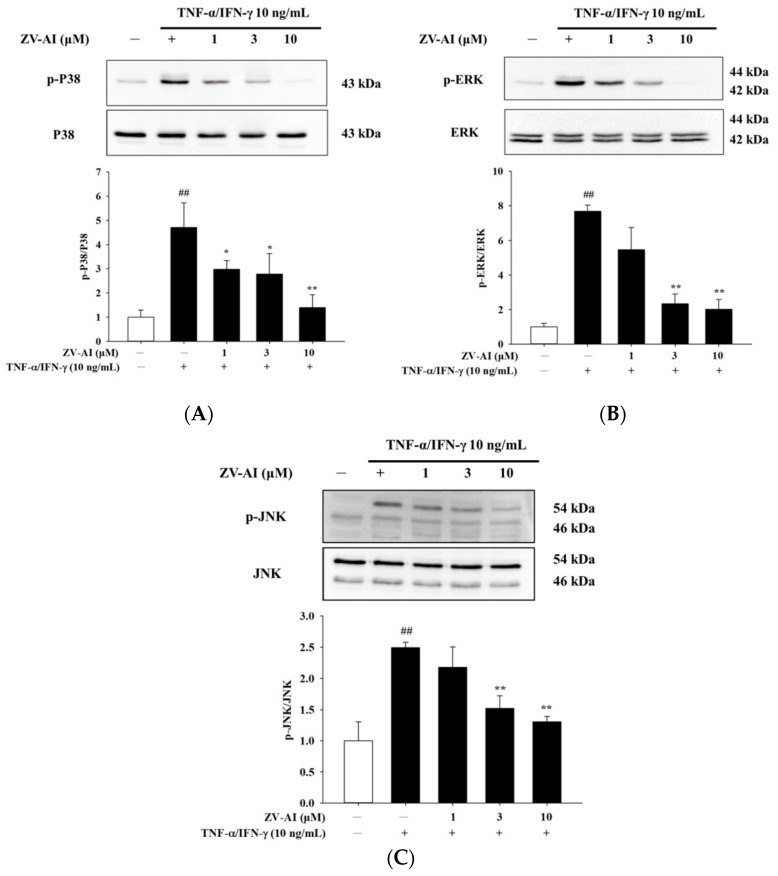
The effect of *epi*-oxyzoanthamine (ZV-AI) on phosphorylation of MAP kinase in TNF-α/IFN-γ-induced HaCaT cells. HaCaT cells were pretreated with different concentrations of *epi*-oxyzoanthamine for 1 h, and then the cells were treated with TNF-α/IFN-γ for 30 min (**A**,**B**) or 1 h (**C**). ## *p* < 0.01 compared with the no-treatment group; * *p* < 0.05 and ** *p* < 0.01 compared with the TNF-α/IFN-γ-induced group.

Many studies have shown that cytokine stimulation of skin cells activates mitogen-activated protein kinase (MAPK) pathway. Therefore, the following experiments will elucidate whether or not *epi*-oxyzoanthamine affects these pathways. First, *epi*-oxyzoanthamine demonstrated no activation or effect on the MAPK pathway (Figure 3). Next, cells were stimulated the cells with IFN-γ and TNF-α for one (or half) hour and found that the pathway to MAPK was activated and phosphorylated (Figure 4). However, the study found that if the cells were pretreated with the *epi*-oxyzoanthamine, the increased phosphorylation was indeed significantly inhibited. This phenomenon was more effective at higher concentrations (Figure 4A–C).

### 2.4. Epi-Oxyzoanthamine Reduced IκB and NF-κB Activation in TNF-α/IFN-γ-Stimulated Keratinocytes

**Figure 5:**
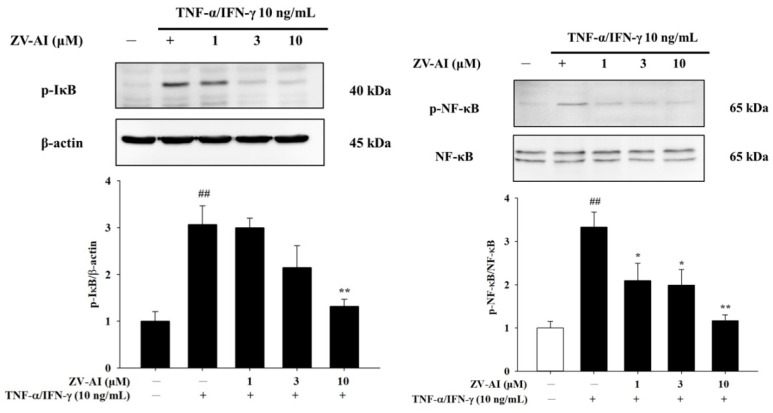
The effect of *epi*-oxyzoanthamine (ZV-AI) on phosphorylation of IκB (**left panel**) and NF-κB (**right panel**) in TNF-κ/IFN-γ-induced HaCaT cells. HaCaT cells were pretreated with different concentrations of *epi*-oxyzoanthamine for 1 h, and then the cells were treated with TNF-α/IFN-γ for 1 h. ## *p* < 0.01 compared with the no-treatment group; * *p* < 0.05 and ** *p* < 0.01 compared with the TNF-α/IFN-γ-induced group.

Previous studies have cited MAP kinase as an upstream NF-κB regulatory kinase [26]. Studies have also found that IFN-γ and TNF-α also increase the phosphorylation of NF-κB and IκB. At the same time, it was found that an increase in the concentration of *epi*-oxyzoanthamine after pretreatment caused an increase in the inhibition of phosphorylation (Figure 5).

### 2.5. The Effect of Epi-Oxyzoanthamine on the Skin Appearance in DNCB-Induced BALB/c Mouse

**Figure 6:**
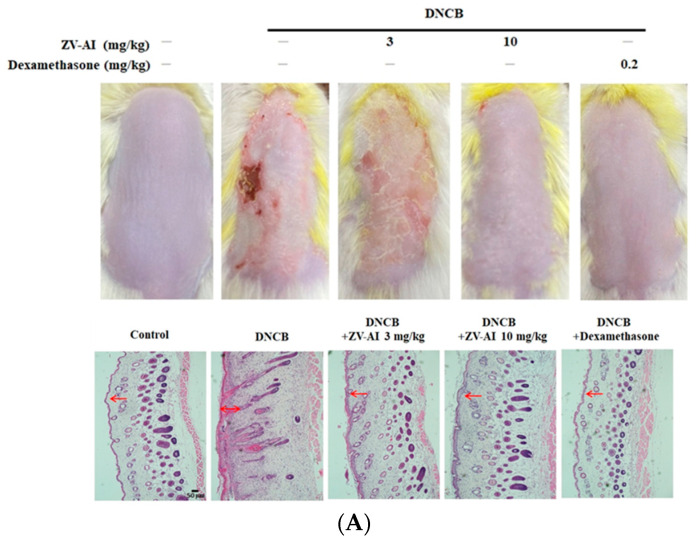

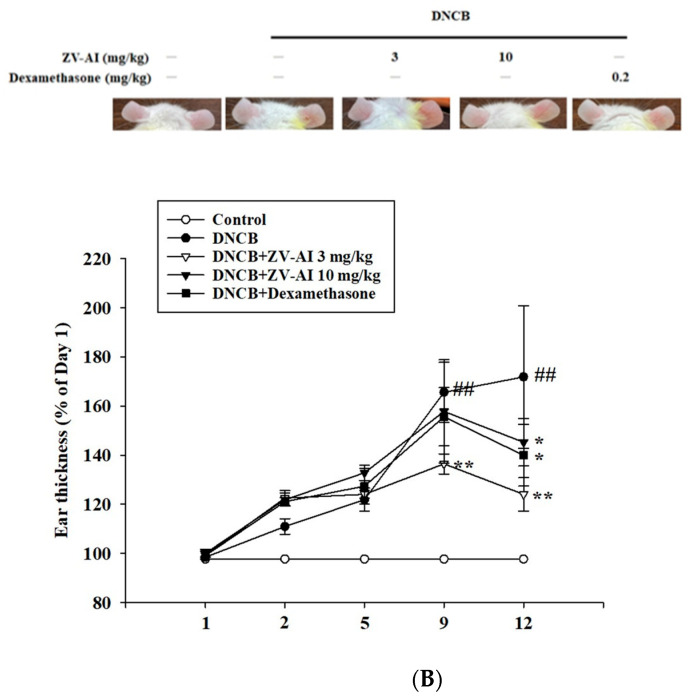
(**A**) Effect of *epi*-oxyzoanthamine (ZV-AI) on DNCB-induced atopic dermatitis in dorsal skin (**upper panel**). Effect of *epi*-oxyzoanthamine on DNCB-induced atopic dermatitis in ears. H and E staining of ear tissue sections. Red arrows point to the epidermis (**lower panel**). (**B**) Upper panel shows the inhibitory effect of the inflammatory response on the ear. Lower panel shows statistical results of ear inflammation thickness. ## *p* < 0.01 compared with the no-treatment group; * *p* < 0.05 and ** *p* < 0.01 compared with the DNCB-induced group.

**Figure 7:**
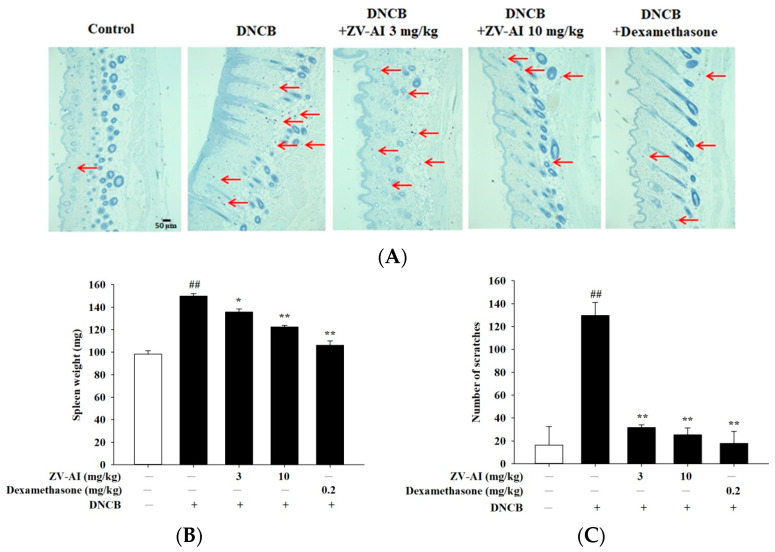
(**A**) Toluidine blue staining of the dorsal skin; scale bar: 50 μm (**upper panel**) and statistical analysis of the mast cells. The red arrow points to the mast cells. (**B**) Effect of *epi*-oxyzoanthamine on spleen weight. (**C**) Analysis of the effect of the change in the number of scratches in BALB/c mice with an atopic-dermatitis-like phenotype. ## *p* < 0.01 compared with the no-treatment group; * *p* < 0.05 and ** *p* < 0.01 compared with the DNCB-induced group.

After the previous in vitro studies, it was found that *epi*-oxyzoanthamine has the potential of anti-atopic dermatitis. Dinitrochlorobenzene (DNCB)-induced contact hypersensitivity (CHS) of the skin in mice is a commonly-used animal model for studying the pathogenesis of contact dermatitis [27]. Given the complexity of factors involved in the pathogenesis of AD, epidermal sensitization with stimuli such as DNCB is a common method used to identify testing drug candidates in AD [28]. When DNCB was applied to the animal’s dorsal skin, it showed atopic dermatitis-like symptoms, such as desquamation and redness, epidermis thickening, skin inflammation, enlarged spleen, and the animal even had scratching behavior (Figure 6 and Figure 7). The above pathological phenomena and symptoms after DNCB pretreatment will be alleviated with the increase of *epi*-oxyzoanthamine concentration. These same preventive effects are also commonly observed after pretreatment with dexamethasone (Figure 6 and Figure 7).

### 2.6. Change in Physiological Functions of DNCB-Induced BALB/c Mouse Skin after Treatment with Epi-Oxyzoanthamine

**Figure 8:**
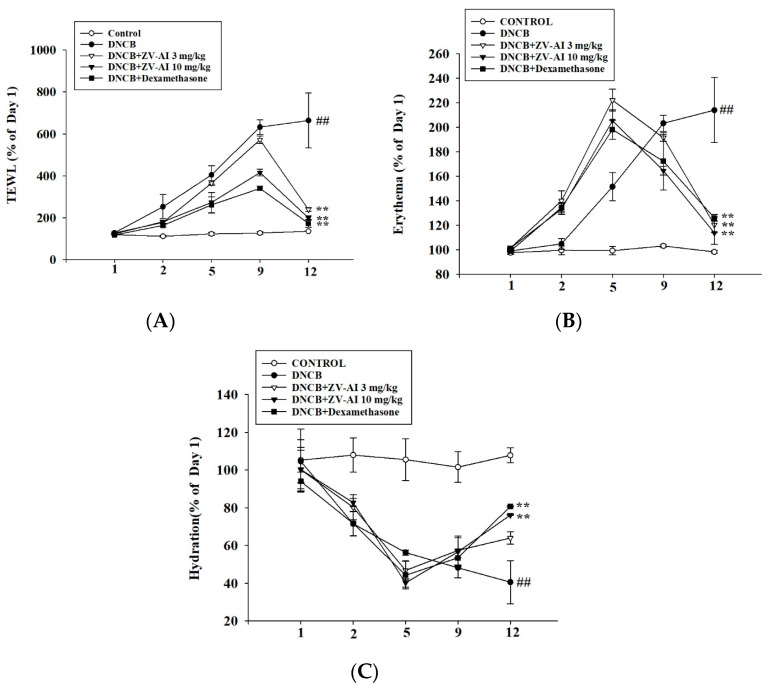
DNCB-induced changes in skin physiological parameters of BALB/c mice after treatment with *epi*-oxyzoanthamine (ZV-AI). Effect of changes in transepidermal water loss (TEWL, (**A**)), erythema (**B**), and hydration (**C**) in the atopic dermatitis-like phenotype of BALB/c mice. Values represent means ± SEM of at least six independent experiments. ## *p* < 0.01 compared to the no-treatment condition; ** *p* < 0.01 compared to the DNCB-induced group.

From the previous pathological sections, *epi*-oxyzoanthamine does have an inhibitory effect on the inflammation caused by DNCB. The physiological values were measured to confirm the efficacy of these effects in improving physiological functions. As shown in Figure 8, improvements were found in physiological function, including transepidermal water loss, skin redness, and skin moisture content, after treatment of *epi*-oxyzoanthamine (Figure 8A–C).

## 3. Discussion

Marine organisms are now a very large source of pharmaceutical resources. Scientists have also discovered many new structures of compounds here, and many structures have even been used to treat diseases. Some of them have even been used clinically or are already in clinical trials. Marine biological sources of these compounds typically include algae, bacteria, fungi, sponges, corals, and other marine animals [29]. Most of their discovered physiological activities are anti-bacterial, anti-viral, anti-tumor, or anti-inflammatory. Among them, the biological activities of sponges, algae, and bacteria have been studied the most, and have shown anti-tumor and anti-bacterial effects [29,30]. There are relatively few drugs from corals [31]. Therefore, our study demonstrates that *epi*-oxyzoanthamine isolated from zoanthid has good anti-dermatological potential, especially in the treatment of atopic dermatitis. These results will open up the application of related compounds produced by corals in the treatment of skin diseases, especially in the treatment of atopic dermatitis.

In previous studies on marine organisms against atopic dermatitis, some studies reported that the red algae—*Pyropia yezoensis*-extract inhibited the production of pro-inflammatory chemokines induced by IFN-γ and TNF-α in HaCaT cells by down-regulating NF-κB [32]. Down-regulation of NF-κB and STAT1 pathways by *Polyopes affinis* suppresses IFN-γ and TNF-α-induced inflammation in human keratinocytes [33]. Red algae—*Sarcodia suiae* sp. ethanol extract has anti-inflammatory effects, alleviates AD symptoms, suppresses inflammatory responses in skin tissue, and restores barrier function in DNCB-induced AD mice [34]. Polysaccharide of brown seaweed—laminarin-topical administration has a protective effect on oxazolone-induced atopic dermatitis-like lesions. Topical application of laminarin can alleviate the overproduction of IgE, mast cell infiltration, and expression of pro-inflammatory cytokines oxazolone-induced atopic dermatitis [35]. Trifuhalol A, a phlorotannin isolated from brown seaweed—*Agarum cribrosum*, inhibited the activations of immune cells and the biosynthesis of cytokines in differentiated B cells and keratinocytes, respectively. Trifuhalol A alleviated pruritus in the Compound 48/80-induced systemic anaphylaxis model and improved symptoms in house dust mite (HDM)-induced AD mice [36]. *Sargassum* polyphenol extract alleviates DNCB-induced atopic dermatitis in NC/Nga mice by restoring skin barrier function [37]. Based on these findings, the effects of marine drugs against atopic dermatitis are attributed to the immunomodulation and regulations of the skin barrier. As shown by the results of this study, *epi*-oxyzoanthamine reduced the cytokines-induced expression of pro-inflammatory cytokines. The mechanisms of action could regulate the map kinase and reduce the action of transcription factor (NF-κB). Therefore, *epi*-oxyzoanthamine could decrease the production of cytokines including IL1β, IL6, and IL8. The appearance of dysfunction of skin barrier is a major symptom in atopic dermatitis patients. Trans-epidermal water loss is an indicator of the skin barrier. Using in vivo studies, it was found that the increase of TEWL-induced DNCB was decreased after treatment of *epi*-oxyzoanthamine. On the other hand, the hydration of the epidermal is increased after the treatment of *epi*-oxyzoanthamine (Figure 8). The erythema and hyperplasia of dorsal skin are the appearance of inflammatory skin. The anti-inflammatory activity of *epi*-oxyzoanthamine was shown in Figure 7 and Figure 8. It was also found that the infiltration of mast cells and the weight of the spleen after treatment of DNCB were decreased after pretreatment with *epi*-oxyzoanthamine. These effects indicate that dysregulation of the immune system could modulate by pretreatment with *epi*-oxyzoanthamine. 

Soft corals are an indispensable source of metabolites with medicinal properties [38]. In the past, many studies have discovered many new compounds with anti-cancer, anti-bacterial, and anti-viral properties in soft corals [39,40,41,42]. Compounds found in soft corals have also been recently reported by many studies for their anti-inflammatory effects. Because inflammation plays an increasingly important role in many clinical diseases [43,44], these compounds are also of particular interest in the application of inflammatory diseases [45,46,47,48]. In recent years, the incidence of diverse chronic inflammatory skin diseases has been increasing, especially atopic dermatitis. Therefore, the discovery of a potent and potential chemical structure of zoanthenamine from zoanthid can be applied to inflammatory skin diseases. In the past, scholars only found that zoanthamines have anti-inflammatory, anti-bacterial, anti-platelet, anti-lymphangiogenesis neuroprotective effects [19,21,49,50]. However, no scholars have proposed the effect on inflammatory dermatitis. Therefore, this is the first to suggest that zoanthamines have anti-inflammatory dermatitis effects. Zoanthamine alkaloids are specific secondary metabolites of marine zoanthids. Importantly, these results show that its ability to resist atopic dermatitis is not inferior to that of components isolated from land plants. Moreover, their mechanism of action is very similar [24,25,51]. Future work will look to identify any unique mechanisms that are used by zoanthamine alkaloids to promote anti-inflammatory dermatitis effects.

## 4. Materials and Methods

### 4.1. Isolation of Epi-Oxyzoanthamine

The lyophilized specimens of *Zoanthus vietnamensis* (1.1 kg) were exhaustively extracted with 95% EtOH to afford 248.2 g of crude extract. An alkaloid-enriched portion of the extract (22.4 g) was subjected to a silica gel column eluted with a stepped hexanes/EtOAc/MeOH (3/1/0 to 0/4/1) to give nine fractions (S1–S9). Fraction S5 (4.9 g) was fractionated by a silica gel column (hexanes/CH_2_Cl_2_/MeOH, 25/10/1 to 0/5/1) to yield six fractions (S5-1 to S5-6). Fraction S5-2 (2.8 g) was applied on a silica gel column (hexanes/acetone, 3/2 to 0/1) to obtain six fractions (S5-2-1 to S5-2-6). Fraction S5-2-3 (491.0 mg) was separated by a silica column (hexanes/acetone, 2/1 to 0/1) to get *epi*-oxyzoanthamine (110.2 mg) [23]. The structure of *epi*-oxyzoanthamine was confirmed by comparing its NMR and MS data with the literature data [52]. The specimens of *Zoanthus vietnamensis* were collected in the northern coastal area of Taiwan, in April 2017. The animal material was identified by Yuan-Bin Cheng [53].

### 4.2. Culture of Human Keratinocyte

The human epidermal keratinocyte line (HaCaT) was used for this study. The HaCaT cell line was provided by Dr. Nan-Lin Wu, Department of Dermatology, Mackay Memorial Hospital, Taiwan. HaCaT was cultured in a 75T flask containing 10% fetal bovine serum (fetal bovine serum, FBS) and Dulbecco’s Modified Eagle Medium (DMEM) with 1% antibiotics (Grand Island, NY, USA). The cells were placed in an incubator at 37 °C and 5% CO_2_, and the cells were allowed to grow to about 80–90% saturation, and then the cells were subcultured.

### 4.3. Western Blotting

Western blots were used to analyze changes in various proteins in cells. Related methods are described in more depth in a previously published paper [24]. HaCaT cells were seeded in 3.5 cm culture dishes. HaCaT cells were seeded in 3.5 cm dishes. After cells reached 90% confluence and were starved for 24 h, they were pretreated with *epi*-oxyzoanthamine for 1 h and then stimulated with TNF-α/IFN-γ for 1 h, respectively. After scraping, cells were crushed by sonication and centrifuged (13,200 rpm, 10 min, 4 °C). After centrifugation, the supernatant was taken, and protein was quantified using the Pierce Protein Assay Kit (Pierce, Rockford, IL, USA). Approximately 20–40 μg of protein was electrophoresed on a 10% SDS-polyacrylamide gel, followed by electroporation with PVDF membranes. After the transfer, the PVDF membrane was placed in a TBS-T solution (Tris-buffered salt/0.05% tween 20) containing 5% nonfat dry milk for 1 h with continuous shaking to avoid nonspecific binding. Then, the PVDF membrane was washed 3 times with TBS-T (30 min in total). After that, the primary antibody was added (diluted 1:1000). PVDF membranes were left overnight at 4 °C and then washed 3 times with TBS-T for 10 min each. Finally, after adding the secondary antibody for 1 h (diluted to 1:1000), the PVDF membrane was washed 3 times with TBS-T, then the developing solution was added, and the membrane was put into the chemiluminescence extraction system (Biostep GmbH Chemiluminescence Imager CELVIN; Type CELVIN^®^S420 FL, Calibre Scientific, LA, USA) for photography.

### 4.4. Real-Time Quantitative Reverse Transcription Polymerase Chain Reaction (RT-qPCR)

HaCaT cells were seeded in 3.5 cm Petri dishes. Cells can reach 90% confluency after 24 h. Cells were pretreated with *epi*-oxyzoanthamine for 1 h and stimulated with TNF-α/IFN-γ for 1 h. Cells were scraped and centrifuged (16,000× *g*, 10 min, 4 °C) and the supernatant was removed. RNA was purified using a total RNA isolation kit (GeneDireX^®^, Vegas, NV, USA)according to the operating procedure of iScript™ cDNA Synthesis Kit (BIO-RAD, Hercules, CA, USA), reagents were added one by one and specified conditions were followed closely to convert RNA into cDNA. Additionally, PowerUp™ SYBR™ Green Master Mix (Applied Biosystems™, Waltham, MA, USA) was used. 7.5 μL ddH_2_O, 2 μL cDNA, 0.25 μL forward and reverse primers, and 10 μL SYBR GREEN were added and mixed well. The relevant primer sequences are listed in Table 1. Finally, RNA was quantified using the ABI StepOnePlus™ Real-Time PCR System (Applied Biosystems™, Waltham, MA, USA).

### 4.5. Animal Model of DNCB-Induced Atopic Dermatitis-like Skin Inflammation

Male mice (BALB/c, 8 weeks old) were purchased from Taiwan National Laboratory Animal Center. The mice were kept in a temperature-controlled and humidity-controlled animal room (controlled at 21 ± 2 °C and 50 ± 20%, respectively). Mice were housed at the Animal Center of Fu Jen Catholic University in Taiwan with controlled laminar filtered airflow and a 12-h light/dark cycle. This experiment was performed after review by the Institutional Animal Care and Use Committee of Fu Jen Catholic University, Taiwan (approval number A10964).

The mice were first divided into four groups: control group, DNCB group, *epi*-oxyzoanthamine (3 and 10 mg/kg) plus DNCB group, and 0.2 mg/kg dexamethasone plus DNCB group. For in vivo experiments, *epi*-oxyzoanthamine was dissolved in dimethylsulfoxide (DMSO), while DNCB was dissolved in 75% ethanol. The former was administered by intraperitoneal injection, while the latter was applied to the skin of the back and right ear in 100 μL and 20 μL, respectively. During the first three days, mice were anesthetized and had their dorsal hair removed. A small magnet (1 mm in diameter and 3 mm in length) was embedded in each mouse’s hind paw. Three days later, the behavior of the mice was observed, and it was ensured that mice were in good physical condition and the skin in the depilated area on the back was normal before starting the experiment. Skin-related physiological values including transepidermal water loss (TEWL), erythema, skin moisture, ear thickness, and number of scratches were measured before the experiment. Photographs were also taken to document changes in the appearance of the skin and ears. The whole experimental process was carried out in a room with constant temperature and humidity. The first phase (days 1–4) is the allergic atopic dermatitis phase. After measuring the physiological value of the mice, 1% DNCB was evenly applied to the skin of the dorsal and right ear. On the fifth day, intraperitoneal injection of *epi*-oxyzoanthamine began. The second phase (days 5 to 14) involved reinduction of atopic dermatitis. We then evenly applied 0.5% DNCB on the back and right ear skin of mice in four experimental groups. The next day, the physiological values of the skin were tested and recorded and photographed. After completing all tests on day 15, mice were euthanized with carbon dioxide (CO_2_) and dorsal skin tissue and spleens were removed for subsequent experimental analysis.

### 4.6. Data and Statistical Analysis

Sigma-Plot software (version 10.0) was used for all statistical analyses of the data. All data are presented as mean ± SEM. Statistical significance was assessed by unpaired two-tailed Student’s *t*-test. *p* < 0.05 was considered significant (* *p* < 0.05, ## and ** *p* < 0.01).

## 5. Conclusions

The results showed that *epi*-oxyzoanthamine significantly decreased skin barrier damage, scratching responses, and epidermal hyperplasia induced by DNCB. It significantly reduced transepidermal water loss (TEWL), erythema, ear thickness, and spleen weight, while also increasing surface skin hydration. These results indicate that *epi*-oxyzoanthamine from zoanthid has good potential as an alternative medicine for treating atopic dermatitis or other skin-related inflammatory diseases.

## Figures and Tables

**Table 1:** Primers used for RT-qPCR.

Name	Forward Primer Sequence (5′–3′)	Reverse Primer Sequence (5′–3′)
IL-1β	CTC TCA CCT CTC CTA CTC ACT	ATC AGA ATG TGG GAG CGA AT
IL-6	ATC AGA ATG TGG GAG CGA AT	GGA CCG AAG GCG CTT GTG GAG
IL-8	ACT GAG AGT GAT TGA GAG TGG AC	AAC CCT CTG CAC CCA GTT TTC
GAPDH	CTG CTC CTG TTC GAC AGT	CCG TTG ACT CCG ACC TTC AC

## Data Availability

Not applicable.
